# Teledentistry use during the COVID-19 pandemic: perceptions and practices of Ontario dentists

**DOI:** 10.1186/s12903-023-02772-y

**Published:** 2023-02-04

**Authors:** Rocco Cheuk, Abiola Adeniyi, Julie Farmer, Sonica Singhal, Abbas Jessani

**Affiliations:** 1 Schulich School of Medicine and Dentistry, Western University, 1151 Richmond St, London, ON N6A 3K7 Canada; 2 Faculty of Dentistry, University of British Columbia, 2199 Wesbrook Mall, Vancouver, BC V6T 1Z3 Canada; 3 Faculty of Dentistry, University of Toronto, 124 Edward St, Toronto, ON M5G 1X3 Canada; 4 Health Promotion, Chronic Disease and Injury Prevention Department, Public Health Ontario, 661 University Avenue, Suite 1701, Toronto, ON M5G 1M1 Canada

**Keywords:** Teledentistry, Practices, Barriers, COVID-19, Ontario, Canada

## Abstract

**Background:**

Teledentistry has demonstrated to expedite oral health consultations, diagnosis, and treatment planning while mitigating COVID-19 transmission risk in dental offices. However, the use of teledentistry by clinicians remains suboptimal. Therefore, this study aimed to determine the perceptions and practices of teledentistry among dentists during the COVID-19 pandemic in Ontario, Canada, and identify associated factors.

**Methods:**

A cross-sectional study using an online 39 item survey was conducted among Ontario dentists in December 2021. The questionnaire inquired about socio-demographic attributes, as well as perceptions of teledentistry use during the pandemic, and its future application. Descriptive statistics including frequency distribution of categorical variables and univariate analysis of continuous variables were conducted. Chi-square test was used determine the associations between professionals’ attributes such as age, gender, years of practice, and location of practice, and respondents use of teledentistry. SPSS Version 28.0 was used for statistical analysis.

**Results:**

Overall, 456 dentists completed the survey. The majority were general dentists (91%), worked in private practices (94%), were between 55 and 64 years old (33%), and had over 16 years of professional experience (72%). Approximately 49.3% reported using teledentistry; 13% started before the pandemic, and 36% during the pandemic. The most common reason for non-utilization was a lack of interest (54%). Respondents identified patient triage, consultation, and patient education as the three most important uses of teledentistry. Female dentists (*p* < 0.05), dentist working in private practice (*p* < 0.05), and those who worked in a single dental office (*p* < 0.05) adopted teledentistry more during the pandemic. Respondents who accessed more resources were more likely to report greater utilization of teledentistry, while those who reported being unconformable with teledentistry (*p* < 0.05) reported less utilization. Additionally, participants who reported feeling comfortable discussing teledentistry with others (*p* < 0.05), were more inclined to use it in the future.

**Conclusions:**

Participants expressed mixed perceptions toward teledentistry with more than half indicating it is reliable for patient triaging and patient follow-ups. Despite the increased utilization during the COVID-19 pandemic, participants' lack of interest in teledentistry emerged as a barrier to its use. More education and knowledge dissemination about teledentistry's areas of application and technical aspects of use can increase interest in this tool, which may lead to a greater uptake by dental professionals.

**Supplementary Information:**

The online version contains supplementary material available at 10.1186/s12903-023-02772-y.

## Background

Teledentistry is a combination of dentistry and tele-virtual communication, which provides patients with dental care at a distance [1]. This approach transitions consultation, diagnosis, and treatment planning away from a dental office environment by utilizing communication technologies such as computers, cameras, and the internet [2]. A commonly used synchronous modality of teledentistry is 'Real-Time Consultation' which involves videoconferencing technology and allows two-way interactions between a dental professional and a patient from different locations [3]. Whereas the asynchronous modality of 'Store-and-Forward Method' involves forwarding remotely monitored clinical information, radiographs, photos, and lab tests to specialists for consultation and treatment planning [4, 5]. Other applications of teledentistry include remote live monitoring of treatment progress when an in-person follow is not feasible, providing preventive care and education programs, and delivering oral healthcare services via mobile applications on phones and tablets (Mobile health) [6, 7].

Previous studies have confirmed the utilization, application, and efficacy of teledentistry [3–5]. A study reports that the implementation of communication technology could result in a substantial improvement in patient care standards [8]. Another study found that the diagnostic accuracy of teledentistry via videoconferencing was comparable to that of in-person examinations [9]. The prominent benefit of teledentistry is allowing dental professionals to treat patients remotely; thereby, reducing the need for long-distance travel [10]. In addition, it has also fostered inter-professional collaboration [2, 11]. However, one obvious limitation of teledentistry is that it cannot assist in the performance of in-clinic procedures [10].

During the recent COVID-19 pandemic, the majority of COVID-19 transmission risks in dental operatories were attributed to aerosol-generating medical procedures (AGMP), which was a major concern in dental settings [12, 13]. Other major concerns included a shortage of Personal Protective Equipment (PPE), the period in which infection prevention and control changes were implemented in clinics during the early stages of the pandemic, maintaining privacy, obtaining informed consent, meeting regulatory requirements for recordkeeping, and the unfamiliarity of using specific codes from fee guides [10].

Consequently, dentists in North America began using virtual modes of teledentistry to provide consultations, assess and triage patients, provide prescriptions, and refer patients to specialists [14]. In the United States, researchers sought to increase patient acceptance of mobile dentistry platforms that use intraoral cameras to provide virtual oral examinations, oral health management, and treatment planning [15]. In Alberta, Canada, teledentistry was used to triage dental patients and determine whether patients’ odontogenic infections could be delayed with drug prescriptions or not during early stages of the pandemic [16]. In British Columbia, Canada, teledentistry was used to establish guidelines for taking acceptable intraoral photography for screening and triaging of potentially malignant oral lesions in home settings as opposed to clinical settings [17].

From late 2020 to June 2022, the dental regulatory body in Ontario Canada (Royal College of Dental Surgeons of Ontario (RCDSO)) recommended that emergency cases be managed via telephone screening risk assessment in which a patient's medical history is gathered and appropriate pharmacotherapies are administered based on the initial assessment [18, 19]. Dental regulatory bodies have also been guiding dentists on using teledentistry to manage emergency dental cases [20]. Even though teledentistry is cost-effective, and efficient in reducing consultation waiting time, the integration and adoption of teledentistry in dental practices in North America and Canada have been challenging [21, 22].

According to the current literature, not fully adopting telehealth was primarily attributed to clinicians' resistance to change and a lack of technological and human resources [23, 24]. Although there is literature that supports patients’ positive perspectives of using teledentistry during the COVID-19 pandemic, a gap exists in understanding the specific factors that influence dentists’ perspectives and willingness to use teledentistry [25]. Therefore, this study sought to bridge this research gap by investigating the perspectives of Ontario dentists on teledentistry before and during the COVID-19 pandemic and identify factors influencing these views. It also sought to determine the types and use of teledentistry by Ontario dentists.

## Methods

### Study design and sample

A cross-sectional study was conducted among dentists in Ontario, Canada, in December 2021. All general dentists and dental specialists licensed to practice in Ontario were eligible to participate. At the time of the study, there were approximately 10,500 practicing dentists and 1,000 dental specialists in Ontario [26, 27]. The minimum sample size for the study was computed as 384 based on an expected prevalence of 50% (no existing study on attitudes to and utilization of teledentistry in Canada), precision level of 5% and level of confidence of 95% [28]. Ethics approval for the study was obtained from the Western University Research Ethics Board (Project ID: 118715).

### Survey tool and data collection

An electronic survey was sent to dentists through Ontario Dental Association listserv to approximately 9500 members representing 90% of dentists registered in Ontario. Prospective participants received a two-week follow-up reminder. The survey comprised a thirty-nine-item questionnaire adapted from previous studies [29–32]. The questionnaire developed in the English language, included items on participants’ sociodemographic and professional characteristics, utilization of teledentistry, current teledentistry practices, and their perceptions of teledentistry (Additional file 1). The questionnaire included mostly close-ended and single-select questions with options for including additional information for some questions. Prior to roll-out, a pilot was conducted on 15 participants and changes were made for comprehension, syntax, and clarity.

### Study variables

#### Socio-demographic and professional characteristics

Information included participants’ socio-demographic (age, gender) and professional characteristics (years of experience, location of practice, population served, primary type of practice, and the number of practice settings). The age groups were categorized as < 34 years, 35–44 years, 45–54 years, 55–64 years, and 65 years and above [33]. The years of experience were grouped as < 6 years, 6–10 years, 11–15 years, and 16 years and above, and further dichotomized for bivariate analysis as 10 years or less and 11 years and above [33]. Location of practice was categorized into three groups (major city, city/town, rural area) based on the population size of the respondents’ primary practice location [34]. Respondents were also asked whether they perceived their practice's neighbourhood to be low-income. They were further asked to identify the number of practices they worked with responses categorized into one, two, or at least three practice settings.

#### Teledentistry utilization and related practices

Participants were asked to indicate their use of teledentistry for clinical practice and when they started to use it. Participants who used teledentistry were asked how often (5-point Likert scale ranging from never to always) they used teledentistry for patient triaging, dental consultations, treatment planning, and were provided with the option to include other teledentistry applications not listed. in addition, they were also asked to indicate their perceived top three most common uses of teledentistry in clinical practice from the same list. Their responses were recategorized into three groups: not selected, ranked unimportant, and ranked important. Therefore, participants were asked to identify their perceived barriers to teledentistry uptake.

#### Perceptions to teledentistry

To assess their perceptions of teledentistry, participants were asked to rate their agreement with twelve items on a five-point Likert scale ranging from strongly agree to strongly disagree. They were further asked to indicate their likelihood of using teledentistry in the future.

### Data analysis

The data was analyzed using SPSS Version 28.0 (SPSS Inc., Chicago, IL, USA)®. Frequency distribution was generated for all variables. Bivariate analyses were conducted to identify factors associated with the participants’ current use and future use of teledentistry. The comparison variables were years of experience, gender, location of practice, type of practice, and type of population served. Chi-squared test was used to test the association between variables. Statistical significance was inferred at *p* < 0.05.

## Results

### Sociodemographic and professional characteristics of study participants

A total of 456 general dentists and dental specialists participated in the current study. Table 1 provides the socio-demographic and professional descriptions of the study participants. Of the 456 participants, the majority identified as general dentists (90.8%), the highest proportion was between 55 and 64 years old (32.9%), and over half were males (57.0%). More than two-thirds indicated they had worked for over 16 years (71.5%), while the majority listed private practice (93.9%) as their primary workplace. Only 14.2% reported they served in a “low-income” neighborhood while 58.6% worked in a major city.

**Table 1:** Summary of socio-demographic and professional characteristics of the study respondents

Variable	N	(%)
*Professional Category*		
General Dentist	414	90.8
Dental Specialist	42	9.2
*Age Category*		
< 35 years	42	9.2
35–44 years	90	19.7
45–54 years	104	22.8
55–64 years	150	32.9
65 years and above	70	15.4
*Gender*		
Female	187	41.0
Male	260	57.0
Prefer not to disclose	9	2.0
*Years of experience*		
< 6 years	36	7.9
6–10 years	47	10.3
11–15 years	47	10.3
16 years and above	326	71.5
*Location of practice*		
Major City	267	58.6
City or town	152	33.3
Remote areas	35	7.7
Unspecified	2	0.4
*Type of practice*		
Private practice	428	93.9
Public health practice	6	1.3
Educational institution	9	2.0
Hospital	6	1.3
Others	7	1.5
*Neighborhood served*		
Low income	65	14.2
Not low income	383	84.0
Unspecified	8	1.8
*Number of practice settings*		
1	341	74.8
2	74	16.2
3 and above	40	8.8
Unspecified	1	0.2
Total	456	100.0

### Usage patterns and perspectives of teledentistry among Ontario dentists

Table 2 shows the prevalence and pattern of teledentistry use among Ontario dentists. A total of 225 (49.3%) respondents reported ever using teledentistry; 60 (13.1%) started before the pandemic and 165 (36.2%) during the pandemic. Regarding the frequency of use during the pandemic (n = 390), 29.2% reported never using it, whereas a small proportion (7.2% and 1.3%) indicated using teledentistry often and always, respectively. For the participants who reported never using teledentistry, the most common reason was a lack of interest (54.1%). Other reasons (31.2%) included billing problems, associates having little control over office administration, and the fact that teledentistry is ineffective. Most respondents (69.7%) cited the dental professional guidelines as their source of information on teledentistry, while 23.0% cited provincial health ministry guidelines. Regarding their level of comfort discussing teledentistry with patients and/or colleagues, the majority (45.6%) indicated they would neither be comfortable nor uncomfortable, while 12.6% would be very uncomfortable and 16.4% uncomfortable. Overall, 18.2% indicated they would be comfortable and 7.2% very comfortable discussing teledentistry with others.

**Table 2:** Teledentistry use among dental professionals

Variable	N	(%)
Use of Teledentistry (n = 456)		
Yes, before pandemic	60	13.1
Yes, during pandemic	165	36.2
No	222	48.7
Unspecified	9	2.0
*Teledentistry modes during the pandemic*^*a*^* (n* = *225)*		
Telephone/mobile phone	51	11.2
Live virtual calls (Zoom, Skype etc.)	13	2.9
Capturing photos from patient’s cell phone	40	8.8
Capturing photos via intra oral cameras	9	2.0
Email containing patient information (photos, charts)	38	8.3
File hosting service (e.g. dropbox, google drive)	4	0.9
Third party platforms (e.g. Dentists online, Turnkey)	2	0.4
*Reasons for not using Teledentistry (n* = *218)*		
No interest	118	54.1
Low awareness of benefit	64	29.3
Privacy concerns	38	17.4
Lack of technological infrastructure	35	16.1
Cost	21	9.6
Other Reasons: (Billing issues, Associates who have no say, “unnecessary”)	68	31.2
*Level of comfort discussing teledentistry (n* = *390)*		
Very comfortable	28	7.2
Comfortable	71	18.2
Neither comfortable nor uncomfortable	178	45.6
Uncomfortable	64	12.6
Very uncomfortable	49	16.4
*Usage frequency during pandemic (n* = *390)*		
Always	5	1.3
Often	28	7.2
Sometimes	97	24.9
Rarely	146	37.4
Never	114	29.2
*Sources of Teledentistry information (n* = *456)*		
Professional association guidelines	318	69.7
Provincial Ministry of Health guidelines	105	23.0
Research articles	28	6.1
Third party sources	21	4.6
Health blogs	12	2.6
Other (e.g. conferences, peers, webinars)	13	2.9
Unspecified	127	27.9

^a^participants selected more than one option in response to the question prompt

Table 3 shows respondents’ opinions about teledentistry. More than half of the respondents (57.9%) agreed that teledentistry was reliable for triaging patients for care and for follow-up care (55.7%). Only 2.6% of the respondents considered technological infrastructure was a barrier to teledentistry utilization. Teledentistry was considered good for co-worker interaction by only 12.3% of the participants. Approximately 27% agreed that it increased access to care and 18.4% considered it improved access to specialist care.

**Table 3:** Participants’ perceptions of teledentistry in clinical practice (n = 456)

No	Statement	Strongly agree/Agree (%)	Neither agree nor disagree (%)	Strongly disagree/Disagree (%)	No response (%)
1	Teledentistry was efficient for referrals during the pandemic	116 (25.5)	172 (37.7)	102 (22.3)	66 (14.5)
2	Teledentistry has improved co-worker interaction during the pandemic	56 (12.3)	182 (39.9)	152 (33.3)	66 (14.5)
3	Teledentistry has improved communication with patients during the pandemic	105 (23.0)	154 (33.8)	131 (28.7)	66 (14.5)
4	Teledentistry was effective in providing oral hygiene instructions	79 (17.3)	120 (26.3)	191 (41.9)	66 (14.5)
5	Teledentistry has improved patient access to oral health care including reducing travel time	125 (27.4)	125 (27.4)	140 (30.7)	66 (14.5)
6	Teledentistry has increased access to specialists for rural and underserved communities	84 (18.4)	197 (43.2)	109 (23.9)	66 (14.5)
7	Teledentistry should be a part of routine dental care	115 (25.2)	88 (19.3)	187 41.0)	66 (14.5)
8	Teledentistry equipment is reliable for dental consultations	181 (39.6)	109 (23.9)	91 (20.0)	75 (16.4)
9	Teledentistry equipment is reliable for triaging patients	264 (57.9)	82 (18.0)	35 (7.6)	75 (16.4)
10	Teledentistry equipment is reliable for dental treatment	59 (12.9)	102 (22.4)	220 (48.2)	75 (16.4)
11	Teledentistry equipment is reliable for patient follow-up	254 (55.7)	83 (18.2)	44 (9.6)	75 (16.4)
12	Teledentistry equipment is reliable for storing and transmitting information safely	132 (29.0)	150 (32.9)	99 (21.7)	75 (16.4)

### Association between participant characteristics and current use of teledentistry

Table 4 shows the association between the participant’s socio-demographic characteristics, professional characteristics, and current use of teledentistry. Gender, type of practice, and number of practice settings in which the dentists worked were all found to be significantly associated with the participants’ current use of teledentistry. Significantly more female dentists (*p* = 0.005) and more respondents who did not work in private practice (*p* = 0.003) adopted teledentistry during the pandemic. Teledentistry use was reported by significantly fewer dentists working in two practice settings (*p* = 0.033). The number of teledentistry resources accessed was also positively associated with current teledentistry use (*p* < 0.001). Similarly, respondents who were uncomfortable discussing teledentistry reported they were not currently using it (*p* < 0.001).

**Table 4:** Association between respondent’s socio-demographic characteristics and current use

Variable (N)	Current use of Teledentistry				
	Started before pandemic (n = 60) 13.2%	Started during pandemic (n = 165) 36.2%	Not using (n = 222) 48.7%	Total (n = 447) 100.0	Chi Sq (*p* value)
*Professional Category*					
General Dentist	13.8	37.2	49.0	90.8	0.922 (0.630)
Dental specialists	9.8	34.1	56.1	9.2	
*Age Category*					
< 35 years	4.9	46.3	48.8	9.2	10.44 (0.235)
35–44 years	20.2	37.1	42.7	19.9	
45–54 years	10.7	35.9	53.4	23.0	
55–64 years	12.5	38.9	48.6	32.2	
65 years and above	15.7	28.6	55.7	15.7	
*Gender#*					
Female	10.3	46.2	43.53.3	41.2	14.95 (0.005)*
Male	15.0	30.3	54.7	56.8	
Others	33.3	33.3	33.3	2.0	
*Years of experience*					
0–10 years	12.3	44.4	43.3	18.1	2.456 (0.296)
11 years and above	13.7	35.2	51.1	81.9	
*Location of practice*					
Major City	11.5	41.4	47.1	58.4	6.335 (0.175)
City or town	16.4	29.6	53.9	34.0	
Remote areas	14.7	35.3	50	7.6	
*Type of practice*					
Private practice	13.8	35.2	51.0	95.3	11.31 (0.003)*
Others	4.8	71.4	23.8	4.7	
*Neighborhood served*^*#*^					
Low income	10.8	41.5	47.7	14.5	0.899 (0.638)
Not low income	13.9	36.1	50.0	85.5	
*Number of practice settings*^*#*^					
1	12.5	33.5	54.0	75.4	10.46 (0.033)*
2	16.9	46.5	36.6	15.9	
3 and above	15.4	48.7	35.9	8.7	
*Number of resources accessed*					
None	14.3	18.5	67.2	26.6	27.31 (< 0.001)*
1	10.9	43.8	45.3	45.0	
2 or more	16.5	43.3	40.2	28.4	
*Level of comfort with discussing teledentistry*^*#*^					
Uncomfortable	4.4	25.6	69.9	29.0	34.67 (< 0.001)*
Neutral	13.5	39.9	46.6	45.6	
Comfortable	20.2	48.5	31.3	25.4	

*statistically significant;

# Valid responses = 390

### Predicting the future usage of teledentistry from participants’ characteristics.

Table 5 shows the association between the participant’s socio-demographic factors, professional attributes, and future teledentistry use. We found statistically significant associations between the type of practice, number of practice settings, level of comfort discussing teledentistry with others, and the participants’ proposed future use of teledentistry. Dentists who are not in private practice (*p* = 0.001) expressed an interest in continuing or beginning to use teledentistry. Dentists working in 3 or more practices indicated an interest in using teledentistry in the future (*p* = 0.007). Participants who reported accessing two or more resources for understanding teledentistry were more likely to use it in the future (*p* < 0.001). Participants who reported they felt comfortable discussing teledentistry with others were also more likely to use it in the future (*p* < 0.001).

**Table 5:** Association between respondent’s socio-demographic characteristics and future use of teledentistry

Variable (N)	Likelihood of future use			
	Yes n = 133 (29.2%)	No n = 323 (70.8%)	Total n = 456	Chi Sq *p value*
*Professional Category*				
General Dentist	119 (28.7)	295 (72.3)	414 (90.8)	0.388 (0.532)
Dental specialists	14 (33.3)	28 (66.7)	42 (9.2)	
*Age Category*				
< 35 years	17 (40.5)	25 (59.5)	42 (9.2)	7.22 (0.125)
35—44 years	29 (32.2)	61 (67.8)	90 (19.7)	
45 – 54 years	31 (29.8)	73 (70.2)	104 (22.8)	
55 – 64 years	33 (22.0)	117 (78.0)	150 (32.9)	
65 years and above	23 (32.9)	47 (67.1)	70 (15.4)	
*Gender*^*b*^				
Female	62 (33.2)	125(66.8)	187 (41.0)	3.91 (0.141)
Male	67 (35.8)	193 (74.2)	260 (57.0)	
Others	4 (44.4)	5 (55.5)	9 (2.0)	
*Years of experience*^*b*^* (N* = *455)*				
0—10 years	30 (36.6)	52 (63.4)	82 (18.0)	2.62 (0.105)
11 years and above	103 (27.6)	270 (72.4)	373 (82.0)	
*Location of practice*^*b*^* (N* = *454)*				
Major City	85 (31.8)	182 (68.2)	267 (58.8)	2.02 (0.364)
City or town	39 (25.7)	113 (74.3)	152 (33.5)	
Remote areas	9 (26.5)	26 (76.5)	35 (7.7)	
*Type of practice*^*b*^* (N* = *454)*				
Private practice	120 (27.8)	312 (72.2)	432(95.1)	9.91 (< 0.001)*
Others	13 (59.1)	9 (40.9)	22 (4.9)	
*Neighborhood served*^*b*^* (N* = *448)*				
Low income	16 (24.6)	49 (75.4)	65 (14.5)	0.94
Not low income	117 (30.5)	266 (69.5)	383 (85.5)	(0.33)
*Number of practice settings*^*b*^* (N* = *455)*				
1	87 (25.5)	254 (74.5)	341 (74.9)	9.74 (0.007)
2	28 (37.8)	46 (62.2)	74 (17.3)	
3 and above	18 (45.0)	22 (55.0)	40 (8.8)	
Number of resources accessed				
None	19 (14.8)	109 (85.2)	128 (28.0)	22.55 (< 0.001)
1	61 (30.4)	140 (69.6)	201 (44.1)	
2 or more	53 (41.7)	74 (58.3)	127(27.9)	
Level of comfort with utilizing teledentistry^b^(N = 390)				
Uncomfortable	11 (9.7)	102 (90.3)	113 (29.0)	71.14 (< 0.001)
Neutral Comfortable	58 (32.5) 64 (58.5)	120 (67.5) 35 (35.5)	178 (45.6) 99 (25.4)	

^b^Non-responses excluded

## Discussion

The study results demonstrated that prior to the COVID-19 pandemic, the use of teledentistry among dentists in Ontario, Canada, was low, but increased during the COVID-19 pandemic. In general, respondents had a mixed view of teledentistry. Our results also revealed that during the pandemic, a greater number of female dentists, dentists working outside of private practices and dentists working in a single dental office began using teledentistry. The results further indicated that dentists working outside of private practices, dentists working in three or more dental offices, dentists who reported accessing two or more teledentistry resources, and dentists who felt comfortable discussing teledentistry with patients and/or colleagues were more likely to use teledentistry in the future.

Approximately half of the participating dentists reported using teledentistry, with the majority starting during the pandemic. Similar trends in the utilization of teledentistry have been reported previously [35, 36], with widespread travel restrictions and lockdowns being a major driver of its use. In addition, the increased uptake of teledentistry is likely due to the support of regulatory bodies and professional associations across Canada such as the RCDSO and Ontario Dental Association [37, 38]. In an effort to promote teledentistry, the Ontario Dental Association introduced a billing code and expanded guidelines for the use of teledentistry in private dental practice during the COVID lockdown. Despite these measures, the implementation of this code faced obstacles, including dentists' lack of awareness of teledentistry codes and the uncertainty of coverage through various insurance companies and their plans, as reported by Singhal and colleagues in 2022 [14]. This has led to a lack of clarity in the compensation process, therefore delaying the optimal implementation of teledentistry [12].

Similar to previous studies, our results showed teleconsultations, triaging, and patient monitoring as being the most common reasons for utilizing teledentistry during the pandemic and was of the highest importance among our participants [30, 39, 40]. In addition to triaging, teledentistry helped reduce the backlog of dental emergencies during the initial lockdown phases, while prioritizing emergency care and preventing the spread of COVID-19 [15]. Teledentistry has also proven to be an important tool in patient education due to its ability to reinforce patient education and hygiene instructions via multiple, reproducible, and personalized follow-ups [41]. A study by Torul and colleagues confirmed that virtual follow-ups via video calls are as reliable as conventional face-to-face follow-ups with the ease of reducing traveling costs and time [42].

In terms of the different modes of teledentistry, audio calls were reported to be the most prefered communication method, followed by sending photos taken from patients’ phones and email communications. In London, UK, Viswanathan et al. also found that a telephone review system was efficient at triaging patients efficiently, especially at the beginning of the pandemic when aerosol-generated procedures were of dire concern [43]. Whereas, in Italy, high-quality photos of simple lesions were regarded as efficient in distinguishing malignant pathologies from potentially malignant pathologies [44]. In contrast to the study conducted by Estai and colleagues in Australia where emails were the most reported mode of communication, our study indicated e-mail to be the third most preferred way of exchanging information with the main advantage of being relatively inexpensive and easy to use. [31]. Without digitalization and phone conversations, the pandemic related lockdowns would have made patient care more challenging [45].

Our findings revealed that disinterest in teledentistry was the most significant barrier to its adoption. This finding was supported by the participants' contradictory attitudes towards teledentistry. For instance, only about a quarter of our respondents agreed that teledentistry would increase access to care, especially by eliminating travel time, indicating low awareness of the benefits of teledentistry. This was an important finding, as research indicates that teledentistry can play an essential role in expanding access to dental care for individuals and communities [46]. It could decrease the number of dental visits for patients, especially for vulnerable groups who have a high risk of complications due to COVID-19 and have higher dental treatment needs such as the immunocompromised and the elderly [46, 47]. According to research, teledentistry has the potential to improve continuity of care, increase preventive interventions, and most importantly, increase access to specialist care and general dentists in rural and remote communities. [48, 49].

More than half of the study participants utilized provincial guidelines for the use of teledentistry. Not surprisingly, respondents who had accessed resources in addition to these guidelines were also more likely to use teledentistry; others reported a similar trend [39]. Evidence suggests that resources such as online training modules by regulatory bodies and continuing education could be linked to successfully implementing teledentistry into routine practice [39, 50]. Our results also identified significant associations between the type of practice setting (private vs. public) and number of practices (one vs. two or more) to be significantly associated with the utilization of teledentistry. Dentists who worked in private practices were less likely to use teledentistry than their counterparts. This underutilization of teledentistry in private practices could be attributed to a lack of interest to invest time and resources in learning and installing new technology [35]. Promotional strategies for integrating teledentistry should be focused on private practitioners and dentists working at multiple practices. Another important tool is to incorporate teledentistry within the undergraduate dental curriculum. Research has shown that educating undergraduate dental students on the integration of teleconsultation, teletriaging, and other important aspects of teledentistry could lead to reductions in clinic costs, travel time, and chair time; thereby, improving patient management and provision of care [44, 51].

Although teledentistry has many advantages, there are also some notable limitations. It is not a substitute for primary dental care but a modality to enhance access to primary dental services [52–54]. Often the quality of care is dependent on the quality of available technology. For instance, the inadequate quality of photographs and videos can impede thorough and careful examination, and follow-ups [55, 56]. Dental professionals who are constantly examining images on electronic devices may develop computer vision syndrome (CVS), which has been linked to symptoms such as eye burning, impaired vision, and neck ache [57]. Lastly, increased usage and storage of data on cloud servers can increase concerns about data privacy and breach [14].

## Limitations

This study examined the attitudes and practices of dentists in Ontario, Canada regarding teledentistry, as well as identified the factors associated with its utilization. The overall sample size is a strength of our study as we attained the estimated minimum sample size. However, the low response rate, which may have been caused by survey fatigue during the pandemic, represents a limitation. Although we have identified factors associated with the utilization of teledentistry, further research is indicated to investigate these factors in depth through qualitative or quantitative methods. Therefore, we are cautious in assuming the generalizability of the study findings [59, 60]. Nevertheless, our findings provide evidence to inform future regulatory decisions and perceived barriers concerning the use of teledentistry.

## Future recommendations

Recent literature has indicated rising popularity of tablets, smartphones, and mobile applications in telehealth due to the adoption of consumer-grade technology expedited by the increased computing power and decreased cost of electronics [61]. This study has focused on assessing the conventional technologies used for teledentistry which can be categorized into the store and forward method, remote patient monitoring, and live video monitoring. Future studies conducted in Ontario elsewhere should include assessing the perceptions and utilization of mobile applications and other advanced technological tools to utilize teledentistry [6, 62].

## Conclusions

Our results concluded that dentists in Ontario, Canada have mixed perceptions about teledentistry. The barriers associated with the underutilization of teledentistry in practice settings include lack of interest and lack of resources. Participants’ characteristics associated with the willingness to use teledentistry post-pandemic include type of practice setting, number of practice settings, and number of resources accessed. Despite the fact that teledentistry can improve access to oral health services and reduce disparities between rural and urban communities, more work is required to improve the clarity and comprehensiveness of federal and provincial guidelines on its integration in private practices. Furthermore, significant reforms are also needed in the undergraduate dental curriculum to integrate and optimize the use of teledentistry.

**Fig. 1:**
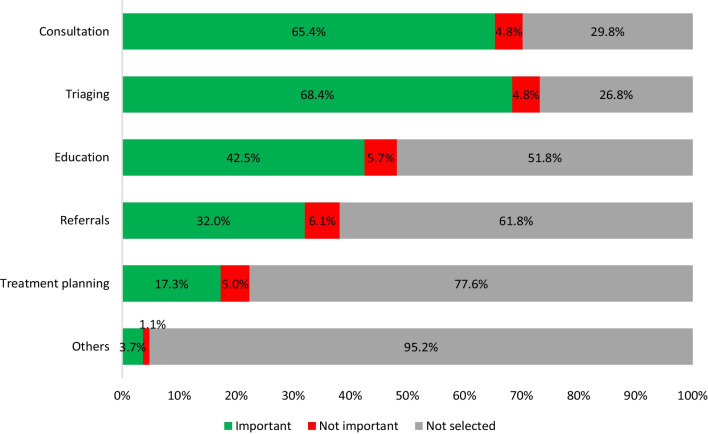
Views on the usefulness of teledentistry in clinical settings. The participant’s ranking of procedures where teledentistry could be useful in clinical practice is shown in Fig. 1. Most participants (68.4%) considered patient triaging an important use for teledentistry in clinical practice followed by consultation (65.4%), educational purposes (42.5%), referrals (32.0%), treatment planning (17.3%) and others (3.7%). Procedures noted within the ‘other’ category included emergency treatment (2.0%), follow-up care (1.3%), rapport building (0.4%)

**Fig. 2:**
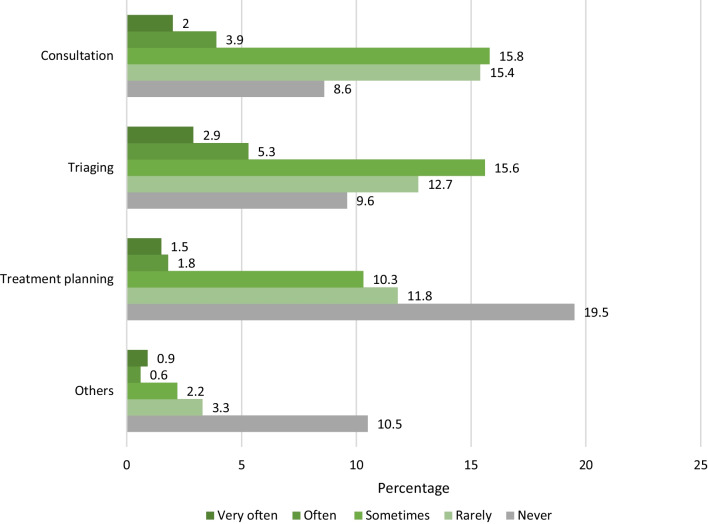
Frequency of teledentistry usage before the pandemic. Figure 2 depicts the usage of teledentistry in clinical practice before the pandemic. Triaging was the most reported (often/always) use for teledentistry (8.2%) followed by consultations (5.9%). Other reported uses included emergency care (1.8%), follow-up after care (0.2%), information and education (0.9%), and referrals (0.4%)

**Fig. 3:**
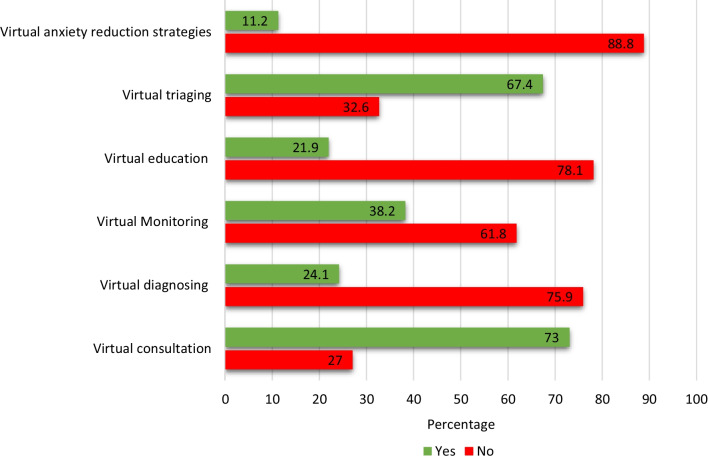
Common procedures conducted using teledentistry during the pandemic. Figure 3 shows the procedures for which the participants reported using teledentistry (n = 178) during the pandemic. Virtual consultation (73.0%) was the most reported procedure, followed by virtual triaging (67.4%), virtual monitoring (38.2%), virtual diagnosing (24.1%), virtual education (21.9%), and only (11.2%) reported using teledentistry for anxiety reduction

**Fig. 4:**
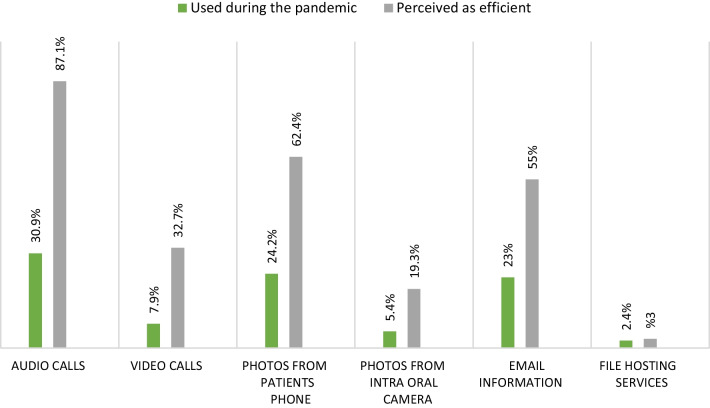
Perceived efficiency of virtual methods and the indicated usage during the pandemic. Figure 4 shows participants’ perceptions on the efficiency and reported use of various teledentistry methods. In general, the number of participants who confirmed using the listed methods was comparatively lower than the number who indicated the method was efficient. For example, although 87.1% agreed audio calls were efficient only 30.9% reported using it during the pandemic. Similarly, while 32.7% agreed video calls were efficient, only 7.9% used it during the pandemic

## Supplementary Information

**Additional file 1.** Appendix A: Questionnaire.

## Data Availability

The dataset used and analyzed during the current study is available from the corresponding author upon reasonable request.

## Abbreviations

AGMP: Aerosol Generating Medical Procedures
PPE: Personal Protective Equipment
RCDSO: Royal College of Dental Surgeons of Ontario
ODA: Ontario Dental Association
