# Supplementary material for: Teledentistry use during the COVID-19 pandemic: perceptions and practices of Ontario dentists
Source: BMC Oral Health. 2023 Feb 4;23:72. doi: 10.1186/s12903-023-02772-y (PMC9899062; doi:10.1186/s12903-023-02772-y)
Supplement: Supplementary file 1 — Additional file 1. Appendix A: Questionnaire. [file 12903_2023_2772_MOESM1_ESM.pdf]

Appendix A: Questionnaire

Q1: Are you a dentist, dental hygienist, or a dental assistant providing clinical care in Ontario?<br><br>a. Yes<br>b. No		
Q2. You identify as ____________ (Gender)		
Q3. Which Local Health Integration Network (LHIN) is responsible for regional administration of public healthcare services in the region you live in?<br><br>a. Erie St. Clair LHN<br>b. South West LHIN<br>c. Waterloo Wellington LHIN<br>d. Hamilton Niagara Haldimand Brant LHN<br>e. Central West LHIN<br>f. Mississauga Halton LHIN<br>g. Toronto Central LHIN<br>h. Central LHIN<br>i. Central East LHIN<br>j. South East LHIN<br>k. Champlain LHIN<br>l. North Simcoe Muskoka LHIN<br>m. North East LHIN<br>n. North West LHIN<br>o. Other: Please Specify		
Q4. Which age group do you belong to? Select one that applies.<br>a. 20-34 years old<br>b. 35-44 years old<br>c. 45-54 years old<br>d. 55-64 years old<br>e. At least 65 years old.		

Q5. Which dental profession do you belong to? **Select one that applies**.<br>a. Dentist<br>b. Dental Specialist<br>c. Dental Hygienist<br>d. Dental Assistant<br>Other ___________________		
Q6. How much work experience in dentistry do you have? (in years) **Select one that applies.**<br>a. 0-5 years<br>b. 6-10 years<br>c. 11-15 years<br>d. ≥16 years		
Q7. How many practice settings do you work at? **Select one that applies.**<br>a. 1<br>b. 2<br>c. 3<br>d. ≥ 4		
Q8. Where do you work the majority of hours? **Select one that applies.**<br>a. Private Practice<br>b. Public Health Practice<br>c. Educational Institution<br>d. Hospital<br>e. Long Term Care Facility<br>Other ____________________		
Q9 What is the location of your primary setting? Select one that applies<br>a. Major city (population over 200,000)<br>b. City or town (population between 15000-200,000)<br>c. Remote Areas (populations less than 15,000)<br>d. Other: Please Specify<br><br>Q9b Is your primary practice in an area that would be defined as "low income neighbourhood"?<br>a. Yes<br>b. No<br>**Definition of Low Income neighborhood: At least 30% of the residents have an after-tax income below 50% of the median after-tax income**		

Q9c In your opinion, please indicate what populations may face challenges to using teledentistry (Select all that apply)<br>a. individuals residing in low income area<br>b. individuals living in rural/remote areas<br>c. elderly population<br>d. indigenous population<br>e. None of the above<br>f. Other: (Please Specify)		
Q10a<br>Since the COVID-19 pandemic, have you incorporated teledentistry into your practice?<br>a. Yes – I was already using teledentistry in my practice<br>b. Yes – I introduced teledentistry during the COVID-19 practice<br>c. No<br><br>Q10b<br>What are the reasons why you have not incorporated teledentistry? Select all that apply:<br>a. I have no interest in using teledentistry<br>b. I am not aware of the benefits of using teledentistry<br>c. Privacy concerns<br>d. Lack of technological infrastructure<br>e. Cost<br>f. Other: (Please Specify)<br><br>Q11a<br>In your opinion, what are the 3 most important usages of teledentistry. Please rank from 1 being the most important to 3 being the least important.<br>a. Consultation<br>b. Patient Triaging<br>c. Patient Education<br>d. Referrals<br>e. Treatment planning<br>f. Others<br><br>Q11b<br>How often have you used any technology for patient triaging, dental consultations, treatment planning and/or case management before the COVID-19 pandemic?		

Patient triaging:<br>a. Never<br>b. Rarely<br>c. Sometimes<br>d. Often<br>e. Very Often<br><br>Dental Consultation:<br>a. Never<br>b. Rarely<br>c. Sometimes<br>d. Often<br>e. Very Often<br><br>Treatment Planning<br>a. Never<br>b. Rarely<br>c. Sometimes<br>d. Often<br>e. Very Often<br><br>Other Purposes: (Please Specify)<br>a. Never<br>b. Rarely<br>c. Sometimes<br>d. Often<br>e. Very Often		

Q12 What resources have you consulted to understand teledentistry? Select all that apply:

a. Guidelines provided by Royal College of Dental Surgeons of Ontario (RCDSO), Ontario Dental Association (ODA), Ontario Dental Hygienists' Association (ODHA), or Ontario Dental Assistant Association (ODAA)
b. Guidelines provided by Ministry of Health
c. Research articles
d. Third-Party Platforms such as Dentist Online, Turnkey Teledentistry, Live Dentist, Denteractive, Smile Virtual, Alpha Dental Excellence
e. Health Blogs
f. Other (Please Specify)

Q13a

Which of the following remote or virtual methods **have you utilized** to support the dental care provided to your patients since the start of the COVID-19 pandemic?

Select all that apply:

~~a.~~ Telephone, mobile phone, or transmitting audio information with patient patients
b. Live virtual video call (such as Skype, Zoom, or Facetime etc.) conducted on a mobile phone, computer, or electronic tablet with patients
c. Capturing photos from patient's cell phone
d. Capturing photos from intraoral cameras

e. E-mail of images, radiographs, charts, reports with other healthcare providers
f. File Hosting Service such as Dropbox, Google Drive to store and share patient information
g. Third-Party Platforms such as Dentist Online, Turnkey Teledentistry, Live Dentist, Denteractive, Smile Virtual, Alpha Dental Excellence etc.
h. Other forms of virtual mediums ________________________________

Q13 b. . Which of the following virtual/teledental procedures do you use in your practice? **<u>Select all that apply</u>**<br>a. virtual consultation (Consult with a patient)<br>b. / virtual diagnosing (Diagnose oral health conditions)<br>c. / virtual monitoring (Monitor a patient's oral health condition)<br>d. / virtual education (Provide oral hygiene education<br>e. / virtual triaging (Triage patients)<br>f. / virtual Anxiety Reduction Strategies<br>g. Other ________________<br>None of the above		

<table>
<tr><td colspan="4">Q14. In your experience, which of the following virtual tools are the most <u>efficient</u> in providing dental care to your patients:

<u>Select all that apply:</u>

<s>i.</s> Telephone, mobile phone, or transmitting audio information with patients
j. Live virtual video call (such as Skype, Zoom, or Facetime etc.) conducted on a mobile phone, computer, or electronic tablet with patients
k. Capturing photos from patient’s cell phone (Asynchronous Collection)
l. Capturing photos from intraoral cameras (Asynchronous Collection)
m. Accessing patient charts including radiographs and pathology reports

n. E-mail of images, radiographs, charts, reports with other healthcare providers
o. File Hosting Service such as Dropbox, Google Drive to store and share patient information
p. Third-Party Platforms such as Dentist Online, Turnkey Teledentistry, Live Dentist, Denteractive, Smile Virtual, Alpha Dental Excellence etc.
q. Other forms of virtual mediums ________________________________
______________________________</td><td></td><td></td></tr>
<tr><td></td><td>Strongly Disagree</td><td>Disagree</td><td>Neither agree nor disagree</td><td>Agree</td><td></td><td>Strongly Agree</td></tr>
<tr><td>Q15. How often have you used teledentistry to monitor a patient’s oral health and/or prognosis since the beginning of the COVID-19 pandemic?</td><td>Never</td><td>Rarely</td><td>Sometimes</td><td>Often</td><td></td><td>Always</td></tr>
</table>

Q16. How comfortable do you feel in explaining aspects of teledentistry to your patients and colleagues?						
Q17. In your experience, how useful are screening methods such as virtual forms, email web forms, or questionnaires conducted over the phone or online platforms for screening patients with COVID- 19 symptoms?	Not useful	Somewhat useful	Moderately useful	Very useful	Extremely useful	
Q18. Do you think teledentistry has been efficient for making referrals during COVID 19 (e.g: to a specialist)?						
Q19. Do you think teledentistry has improved the interaction between coworkers within a practice during the COVID-19 pandemic?						
Q20. Do you think teledentistry has improved your communications with patients during the COVID-19 pandemic?						
Q21. Do you think teledentistry is effective in providing oral hygiene instructions to patients?						
Q22. Do you think teledentistry has improved patient access to oral healthcare during COVID-19 pandemic including reducing unnecessary travel time?						
Q23. Do you think teledentistry has increased access to specialists for rural and underserved communities?						
Q24. Do you think teledentistry should be a part of routine dental care?						
Q25. In your opinion, please indicate if teledentistry has been helpful in providing any of the following **routine non-urgent dental procedures** during the COVID-19 pandemic?<br><br>**Examples of Level-1 dental care:**<br>- complete oral exam<br>- full mouth x-rays	Not at all helpful	Slightly Helpful	Moderately Helpful	Very helpful	Extremely helpful	

- recall examinations<br>- bitewing x-rays<br>- routine diagnostic and laboratory procedures<br>- one unit of light scaling and polishing<br>- fluoride treatments<br>- oral hygiene instruction<br>- space maintainers<br>- fillings (amalgam, silicate, acrylic, and composite), retentive pits , and pit and fissure sealants<br>- pre-frabricated full coverage restorations<br>- minor surgical procedures, simple extractions<br>- complicated extractions including impacted and residual roots<br>- consultations, anesthesia and conscious sedation<br>- denture repairs, relines and rebases<br>- injection of antibiotic drug.						
Q26. Do you think teledentistry has been useful in helping dental professionals provide **emergency care** during the COVID-19 pandemic?<br><br>**Definition of emergency care**: Treatment that would resolve oral-facial trauma, cellulitis especially if it compromises patient's airway, prolonged bleeding, or pain that cannot be managed by over-the-counter medications						
Q27. Do you think teledentistry has been useful in helping dental professionals provide **urgent care** during the COVID-19 pandemic?						

**<u>Definition of urgent care:</u>** Treatment of conditions that would require immediate attention to relieve pain or risk of infection which includes but is not limited to:<br>- severe dental pain from pulpal inflammation<br>- pericoronitis/ third-molar pain<br>- surgical post-operative osteitis, dry socket dressing changes<br>- abscess or localized bacterial infection resulting in localized pain and swelling<br>- tooth fracture resulting in pain, pulp exposure or causing soft tissue trauma<br>- extensive caries or defective restorations causing pain<br>- dental trauma with avulsion/luxation<br>- final crown/bridge cementation if the temporary restoration is lost, causing gingival irritation<br>- biopsy of suspicious oral lesion<br>- replacing temporary filling in an endodontic access opening<br>- snipping or adjusting orthodontic wire or appliance piercing or ulcerating oral mucosa<br>- suture removal<br>- dental adjustments or repairs when function is impeded						
Q28d In rural area, do you think that a major challenge in delivering teledentistry is a lack of technological infrastructure (infrastructure with clinic, general infrastructure in the community or both)?<br><br>THIS QUESTION WILL ONLY DISPLAY IF PARTICIPANT CHOOSES "REMOTE AREA" in QUESTION 9.						

Q29. Do you have concerns about confidentiality or privacy of your patient data while utilizing online platform/s for patient management within your dental practice?	Not at all concerned	Slightly concerned	Somewhat concerned	Moderately concerned		Extremely concerned
Q30. Do you think teledentistry equipment (telephones, intraoral cameras, cell phones, electronic tablets, laptops, desktops etc.) are reliable for patient treatment and management?						
Q31a. In the future, will you continue using teledentistry after the COVID-19 pandemic has passed?<br><br>THIS QUESTION WILL ONLY APPEAR IF PARTICIPANT HAS ANSWERED “YES” to QUESTION Q10a						
Q31b. In the future, will you start using teledentistry or incorporate elements of teledentistry in your work?<br><br>THIS QUESTION WILL ONLY APPEAR IF PARTICIPANT HAS ANSWERED “NO” to QUESITON Q10a						
Q32. Do you think you have received adequate and informative support guidelines from your regulator/association such as Royal College of Dental Surgeons of Ontario (RCDS), College of Dental Hygienists of Ontario (CDHO) Ontario Dental Association (ODA), Ontario Dental Assistants Association (ODAA), or Ontario Dental Hygienists’ Association (ODHA) regarding the utilization of teledentistry?						
